# Traditional Chinese medicine on treating premature ejaculation: A systematic review and meta-analysis

**DOI:** 10.1097/MD.0000000000015379

**Published:** 2019-05-03

**Authors:** Yongqiang Li, Yunyun Duan, Xudong Yu, Jisheng Wang, Zeyu Yao, Xihao Gong, Xiaoyong Gong, Wei Zheng, Yahui Xue, Jianen Guo

**Affiliations:** a Department of Andrology, The Second Affiliated Hospital of Shaanxi University of Traditional Chinese Medicine, Shaanxi; b Department of Andrology, Dongzhimen Hospital, Beijing University of Chinese Medicine, Beijing; c Academy of Traditional Chinese Medicine, Chengde Medical University, Heibei, China.

**Keywords:** premature ejaculation, protocol, systematic review, traditional Chinese medicine

## Abstract

**Background:**

Premature ejaculation is a form of male sexual dysfunction. As people's lifestyle changes and the population ages, the incidence of premature ejaculation continues to increase. Many clinical trials have proven that Chinese medicine has a significant effect in the treatment of premature ejaculation. In this systematic review, we aim to evaluate the effectiveness and safety of Traditional Chinese medicine for premature ejaculation.

**Methods:**

We will search for PubMed, Cochrane Library, AMED, EMbase, WorldSciNet; Nature, Science online and China Journal Full-text Database (CNKI), China Biomedical Literature CD-ROM Database (CBM), and related randomized controlled trials included in the China Resources Database. The time is limited from the construction of the library to April 2019. We will use the criteria provided by Cochrane 5.1.0 for quality assessment and risk assessment of the included studies, and use the Revman 5.3 and Stata13.0 software for meta-analysis of the effectiveness, recurrence rate, and symptom scores of premature ejaculation.

**Ethics and dissemination:**

This systematic review will evaluate the efficacy and safety of Traditional Chinese medicine for treating premature ejaculation. Because all of the data used in this systematic review and meta-analysis has been published, this review does not require ethical approval. Furthermore, all data will be analyzed anonymously during the review process Trial.

**Trial registration number:**

PROSPERO CRD42017065316

## 1 Introduction

Premature ejaculation (PE) is a form of male sexual dysfunction. Definitions of PE consider the time to ejaculation, the inability to control or delay ejaculation, and the negative consequences of PE.^[1,2]^ One widely used definition is the persistent or recurrent ejaculation with minimal sexual stimulation before, on, or shortly after penetration and before the person wishes it. PE can be either lifelong (primary) or acquired (secondary). Lifelong PE is that which has been present since the person's first sexual experiences, while acquired PE is that which begins later following normal ejaculation experiences.^[3–5]^ PE may occur secondary to another condition such as erectile dysfunction or prostatitis. Men with PE are more likely to report lower levels of sexual functioning and satisfaction, and higher levels of personal distress, and interpersonal difficulty than men without PE.^[6,7]^ They may also rate their overall quality of life lower than that of men without PE. In addition, the partner's satisfaction with the sexual relationship has been reported to decrease with increasing severity of the man's condition. Surveys in the UK, the USA, and other countries suggest that PE is the most common male sexual dysfunction, with prevalence rates of 18% to 31%.^[8–10]^ The treatment of PE should attempt to alleviate concern about the condition as well as increase sexual satisfaction for the patient and the partner.

Treatments for PE mainly include drug therapy and psychological and behavioral therapy.^[11]^ Oral 5-HT receptor reuptake inhibitors (SSRIs) are well-established and effective therapies for the treatment of PE, including fluoxetine,^[12]^ paroxetine,^[13]^ sertraline,^[14]^ dapoxetine,^[15]^ and so on. However, the side effects of SSRIs, such as nausea, vomiting, and dry mouth are somewhat confusing for clinicians.^[16]^ At the same time, evidence shows that the efficacy of psychological and behavioral therapy is also not clear.

As the most important part of Chinese medicine, Chinese herbal medicine has been widely used in clinical trials of PE in recent years. Chinese medicine believes that the cause of PE is mainly in the kidney.^[17,18]^ Yin deficiency and heat, qi stagnation, and blood stasis are its main pathogenic factors. Through the application of traditional Chinese medicine in the treatment of PE's unique diagnosis and treatment system, clinical efficacy is significant.^[19,20]^ Modern research has shown that effective active ingredients in traditional Chinese medicine can improve the blood supply of peripheral blood vessels and achieve therapeutic purposes.^[21]^ Through the action mechanism of multi-faceted and multi-target, TCM regulates the body function as a whole and has unique advantages in the treatment of PE.

In the preliminary searches of the electronic databases, we found that randomized controlled trials (RCTs) of Traditional Chinese medicine for PE are on the rise.^[22,23]^ However, due to the limitation of the size and number of clinical centers, most clinical trials are small samples with low-quality and lack of evidence-based exploration. Besides, the publication of the similar systematic review has not been retrieved in the database. Therefore, this review hopes to adopt meta-analysis to evaluate the efficacy and safety of acupuncture in the treatment of PE and provide evidence for its application in clinical practice.

## 2 Methods

This is a systematic review and ethical approval was not necessary.

### 2.1 Study registration

This systematic review protocol has been registered on PROSPERO as CRD42017065316. (http://www.crd.york.ac.uk/PROSPERO/display_record.php?ID=CRD42017065316).

### 2.2 Eligibility criteria

#### 2.2.1 Type of study

Take TCM or Chinese medicine combined with other effective interventions as main treatment, including randomized controlled trials of the control group (effective methods other than traditional Chinese medicine). Language is limited in Chinese and English. Non-randomized controlled trials, quasi-randomized controlled trials, case series, case reports, and crossover studies will be excluded.

#### 2.2.2 Participants

The cases included are adult male patients over 18 years old who have diagnosed PE. The region, nation, ethnic, and sources are not limited.

#### 2.2.3 Types of interventions

##### 2.2.3.1 Experimental interventions

The drug composition, the dose-specific Chinese medicine preparation, or the combined Western medicine are used as experimental interventions. Both prescription and Chinese patent medicines will be included. Other traditional Chinese medicine treatments such as intravenous medication, acupuncture, and massage will be limited.

##### 2.2.3.2 Control interventions

As for the control interventions, who accepted simple Western medicine can be used as a control intervention or didn‘t get any treatment as a blank control would be adopted. However, once they had accepted the therapy of TCM, the trials will be rejected.

#### 2.2.4 Outcomes

##### 2.2.4.1 Primary outcomes

The primary outcome measurement will be IELT.

##### 2.2.4.2 Secondary outcomes

We also need to pay attention to the following outcomes: premature ejaculation diagnostic tool (PEDT), Arabic index of premature ejaculation (AIPE), and index of premature ejaculation (IPE). More importantly, the adverse reactions of patients during medication will also be taken seriously.

#### 2.2.5 Data source

##### 2.2.5.1 Electronic searches

Database Search: PubMed, Cochrane Library, AMED, EMbase, WorldSciNet, Nature, Science online and China Journal Full-text Database (CNKI), China Biomedicalstudies CD-ROM Database (CBM), China Resources Database. A studies review of clinical studies on acupuncture (or acupuncture) for the treatment of premature ejaculation published in domestic and foreign biomedical journals from the establishment of the library to April 2019. Based on the standards of the Cochrane Collaboration Workbook of the International Evidence-Based Medicine Center, a manual and computer-based approach is used to conduct relevant studies searches. Search terms include: Chinese medicine, traditional Chinese medicine, proprietary Chinese medicine, Chinese herbal medicine, PE, and sexual dysfunction. The complete PubMed search strategy is summarized in Table 1.

**Table 1:**
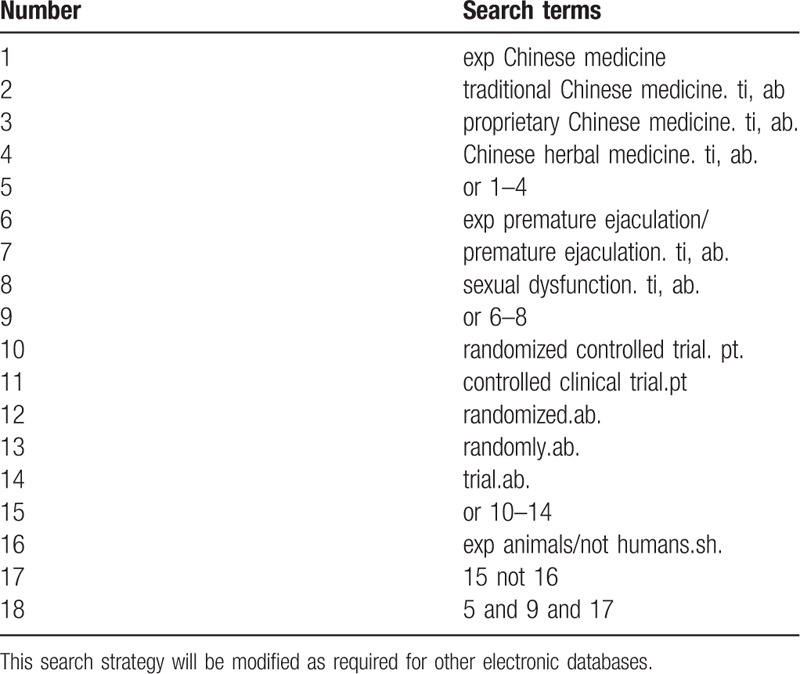
Search strategy used in PubMed database.

##### 2.2.5.2 Searching other resources

The manual search is mainly for dissertations, ongoing experiments, and grey literature. We will look for abstracts of dissertations, conference papers, and conference papers related to acupuncture and PE. Ongoing trials for the new reviews that are relevant to this term will be retrieved from the WHO International Clinical Trials Registry Platform (ICTRP), ClinicalTrials.gov, and the Chinese Clinical Trial Registry. For ongoing experiments, we will try to contact the trial author to help provide up-to-date clinical data. Potential gray literature will be elected in OpenGrey.eu. website.

##### 2.2.5.3 Data collection and analysis

**Figure 1:**
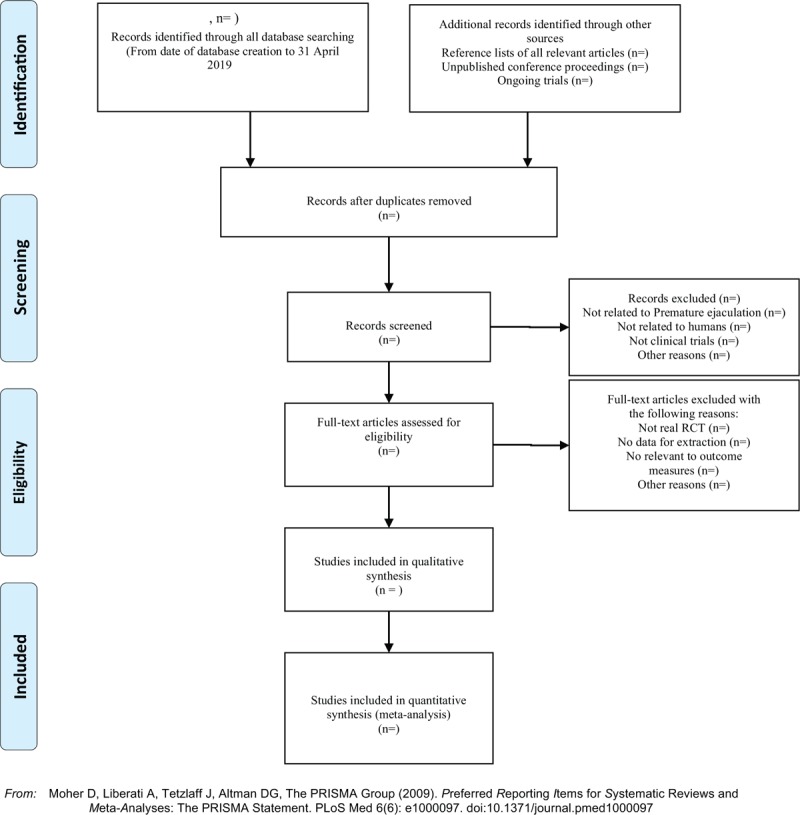
The PRISMA flow chart.

Applying the Endnote X7 software to manage the included references. Two qualified evaluators independently screened the titles and abstracts of the selected studies, excluding duplicates and documents that did not significantly conform to the study. After a preliminary evaluation, the selected documents will be read one by one. Exclusions were based on inclusion criteria for uncontrolled studies, no randomization, inconsistent assessment criteria, and similar data. If there are different opinions, the third reviewer should be consulted. Studies information and data extraction were carried out on the final included studies, including the experimental methods of the study, the basic information of the included cases, the observation period, the intervention methods, observation indicators, and test results of the treatment group and the control group. The details of selection process will be shown in the PRISMA flow chart (Fig. 1).

##### 2.2.5.4 Risk of bias

The quality of the studies will be assessed by using the Cochrane Handbook 5.1.0 (Cochrane Handbook 5.1.0). The assessment will include random sequence generation, randomization correctness, allocation scheme hiding, blinding of patients and implementers, accuracy of data results, and other risk of bias. The risk of low bias is expressed as “low risk” and the risk of high bias is expressed as “high risk.” The information provided in the studies is inaccurate or does not provide sufficient information for the bias assessment to be expressed as “unclear risk.” The above content evaluation was independently evaluated by 2 researchers, and any differences will be resolved through discussions with the third reviewer.

##### 2.2.5.5 Statistical analysis

The meta-analysis in this study will use Rev Man 5.3 and Stata 13.0 statistical software. Heterogeneity tests will be used for the included experimental studies. The numerical variable will be expressed as the normalized mean difference (SMD) with a confidence interval (CI) of 95%. The heterogeneity of each pairwise comparison will be tested by chi-square test (test level *α* = 0.1). If there is no heterogeneity, a fixed effect model will be used. If there is significant heterogeneity between a set of studies, we will use a random effects model (REM) for meta-analysis. We will explore the reasons for the existence of heterogeneity from various aspects such as the characteristics of the subjects and the degree of variation of the interventions. The source of heterogeneity is further determined by means of sensitivity analysis.

##### 2.2.5.6 Publication bias

If a result of a meta-analysis contains >10 articles and above, we will use a funnel plot to test the risk of publication bias. Quantitative methods such as Begg testing and Egger testing will be used to help assess publication bias in the application.

##### 2.2.5.7 Quality of evidence

The GRADE method will be used to assess the quality of evidence for key outcomes. This assessment will be conducted through a Guideline Development Tool. (GRADEpro GDT, https://gradepro.org/).

## 3 Discussion

In recent years, with the changes in people's lifestyles and the aging of the population, the incidence of premature ejaculation has increased.^[24]^ Traditional Chinese medicine believes that the etiology and pathogenesis of premature ejaculation is related to spleen and kidney deficiency and qi and blood block. Traditional Chinese medicine can play the role of strengthening the spleen and kidney, promoting blood circulation and collaterals, and at the same time can improve the mood and achieve the purpose of treatment.^[25]^ From the modern medical point of view, some active ingredients in traditional Chinese medicine can not only improve the blood supply of peripheral blood vessels, but also play a corresponding therapeutic role.^[26]^ With the deepening of understanding of PE, the trials and clinical reports of traditional Chinese medicine for PE have gradually increased. Whether it is syndrome differentiation or special disease, Chinese medicine has achieved good results in the treatment of PE. To the best of our knowledge, there has been no comparison of the efficacy and safety of traditional Chinese medicine in the treatment of PE in recent years. Therefore, we will compare the effectiveness and safety of traditional Chinese medicine in the treatment of PE with systematic evaluation and meta-analysis. The results of this study can provide a possible ranking for the treatment of PE by Chinese medicine. We hope that the results will provide clinicians with the best options for treating PE and provide research directions. Although we will conduct a comprehensive search in this study, languages other than Chinese and English will be restricted, which will lead to some bias. In addition, the relevant literature on the treatment of PE in Chinese medicine is small and the overall quality is low, which may affect the authenticity of this study. Therefore, we hope that in the future, we will have a more rigorous and reasonable multi-center randomized controlled trial to explore the clinical efficacy of traditional Chinese medicine in the treatment of PE, so that the conclusion is more objective and reasonable.

## Author contributions

**Data curation:** Yongqiang Li, Xudong Yu, Jisheng Wang.

**Formal analysis:** Yongqiang Li, Yunyun Duan, Xudong Yu, Jisheng Wang.

**Funding acquisition:** Yunyun Duan, Jisheng Wang.

**Investigation:** Jisheng Wang, Zeyu Yao.

**Project administration:** Xihao Gong, Yongqiang Li, Yunyun Duan.

**Resources:** Xihao Gong.

**Software:** Xiaoyong Gong.

**Supervision:** Xiaoyong Gong, Wei Zheng, Xudong Yu.

**Validation:** Zeyu Yao, Xihao Gong

**Writing – original draft:** Wei Zheng, Jianen Guo.

**Writing – review & editing:** Wei Zheng, Yahui Xue, Jianen Guo.
